# The role of midwives in first‐trimester abortion care: A 40‐year experience in Tunisia

**DOI:** 10.1002/ijgo.13010

**Published:** 2020-07-31

**Authors:** Selma Hajri, Hedia Belhadj

**Affiliations:** ^1^ Groupe Tawhida Ben Cheikh Tunis Tunisia

**Keywords:** Abortion care, Medical Abortion, First trimester, Health worker roles, Midwives, Safe abortion, Task‐sharing, Tunisia

## Abstract

**Objective:**

To review the relevant literature on abortion and summarize interviews with key stakeholders to assess the role of midwives in the evolution of abortion‐related care in Tunisia.

**Methods:**

Interviews with eight stakeholders from different organizations based on a guide developed for the study, focusing on policies, strategies used for implementation, capacities used for expansion, user opinions and experience, obstacles and facilitators, and control and evaluation.

**Results:**

Task‐sharing for midwifes was encouraged in the family planning program from the beginning and when medical abortion was introduced. It allows midwifes to contribute widely, develop good skills and performance for several tasks, and helps reduce regional disparities in human resource allocation. Success and safety of home use of medical abortion confirms the ability of women to manage their own abortion. Yet, obstacles to accessing abortion still exist for several reasons.

**Conclusion:**

This study, based on interviews with personnel with significant experience and solid knowledge of sexual and reproductive health services, allowed us to consider proposals for a future strategy to integrate task‐sharing into abortion care and address the barriers to legal and safe abortion access for all women in Tunisia.

## Synopsis

Task‐sharing of abortion care with midwives has a positive impact on quality of services and addresses barriers to accessing abortion in Tunisia.

## 1 Introduction

Tunisia is a small country of 11 million inhabitants in north Africa. It is the only country in the Middle East and North Africa region where abortion is legal during the first 3 months of pregnancy at a woman’s request.^1^ The law that legalized abortion was instituted as part of a political strategy to modernize Tunisian society. Just a few months after Tunisian independence from France in 1956, a Personal Status Code was promulgated, granting women more rights in several areas—particularly marriage (e.g. divorce, age of marriage) and in relation to male family members (e.g. parental authority, certain inheritance rights)—by expanding the existing laws that were strictly based on Islamic Law, particularly the Maliki and Hanafi schools of law. These developments were followed shortly after by the implementation of policies that guaranteed compulsory education for boys and girls.

Following the results of the national censuses carried out after 1957 and the first experimental program of family planning conducted in 1964–65^2^, ^3^ with support from the Ford Foundation, Tunisia adopted a population policy supported by a series of legal measures:

1. Fixing the minimum age of marriage at 17 years (1959) in the Personal Status Code^1^;
2. Adopting the Family Planning Program (1965);
3. Legalizing abortion in the first trimester at a woman’s request (1973); and
4. Creating the National Office of the Family and Population (ONFP) (1973).^4^

Tunisia’s population policy was also supported by a series of comprehensive programs that were developed in conjunction with other measures based on the Personal Status Code.

Abortion became integrated as part of the national family planning program. The abortion law adopted in 1965 determined that abortion, which was then performed by dilatation and curettage (D&C), be carried out under certain conditions, namely by a physician, in a public or private authorized institution, and only for women who already had more than five children. In 1973 the law was amended further, allowing voluntary termination of pregnancy until 14 weeks at the woman’s request and therapeutic termination of pregnancy after 14 weeks.

The advanced progress of the national family planning program, in which abortion became an integral part, was based on a proactive policy supported by state involvement at the highest level. It included increases in budget allocations, investment in human resources and mobilization, and national support. The program also expanded family planning services in national health facilities (a clinic in each of the 24 governorates and family planning services in each of the 13 tertiary hospitals); deployed 13 mobile clinics (up from 16 in 1974 to 50 in 1979^5^); mobilized international financial and technical resources (e.g. technical support and contraceptive products for contraception contributed by USAID); and garnered support from national organizations, including women’s organizations such as the National Union of Women of Tunisia, the first national women’s defense organization, and local and national networks of volunteers for the dissemination of information on contraception, abortion, and prevention of sexually transmitted infections (STIs).^5^

In the 1990s, coinciding with the program of the International Conference on Population and Development (ICPD), national family planning shifted from population planning to a rights‐based approach, including providing access to family planning and abortion as part of women’s reproductive rights.

The number of health workers increased significantly during the decade (2005–2015). Furthermore, increasing the number of midlevel providers was one of the objectives of the government (Table 1) with the development of scholarships and universities.

**Table 1:** Health workers in Tunisia.^a^

Health worker	2001	2005	2010	2015	No. per 10 000
Doctors	7767	9422	12 996	14 507	13.2
Public sector	4327	4727	6723	6832	6.2
Private clinic	3440	4695	6273	7675	6.9
Paramedical personnel (midwife/technician/anesthetist/kinesiotherapist	27 127	29 607	34 195	39516	35.9
Senior technicians	7284	8677	10 359	12 307	11.2
Nurses	19 843	20 930	23 836	27 209	24.7

^a^ Source: INS.^8^

During the 2000s, decreases in total fertility rate and maternal and infant mortality, as well as an increase in life expectancy were evident, as well as increased access to education for girls and greater opportunities for employment for women.^6^ The World Health Organization (WHO) reported that the maternal mortality ratio (MMR) decreased from 131 per 100 000 live births in 1990 to 62 per 100 000 live births in 2015.^7^ Furthermore, the 2008 National Maternal Mortality Survey revealed an MMR of 44.8 per 100 000 live births, and the rate of deliveries performed by qualified staff (including midwives and nurses) increased from 76.3% in 1990 to 97.6% in 2013.^8^ Demographic indicators also improved (Table 2), with a birth rate of 17 live births per 1000 population and a total fertility rate of 2.05 in 2012.^7^ In 2015, the MMR reported in a national survey was estimated to be 39 per 100 000 live births,^8^, ^9^ against a Millennium Development Goal target of 18.7 per 100 000 live births.^10^, ^11^

**Table 2:** Demographic indicators in Tunisia.^a^

Demographic indicator	2007	2009	2010	2011	2014
Birth rate (live births per 1000 population, per year)	17.5	17.7	18.6	18.8	19.5
Fertility rate (births per 1000 fertile women, per year)	1.19	1.20	1.29	1.29	1.4
Fecundity index	2.00	2.05	2.13	2.15	2.20
Prevalence of contraception (% of women aged 15–45 years using contraceptives)		62			58

^a^ Source: INS.^8^

**Figure 1:**
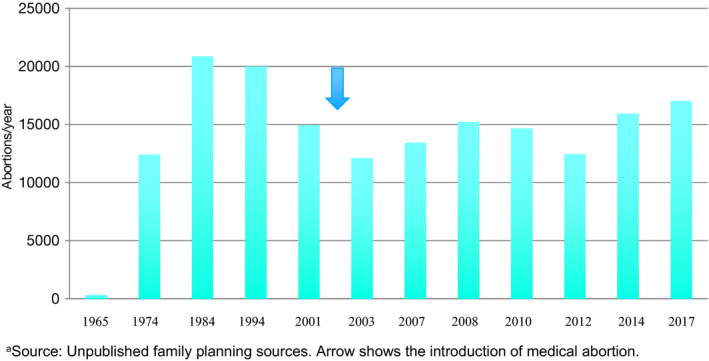
Abortions performed in the public health sector. Source: Unpublished family planning sources. Arrow shows the introduction of medical abortion.

With the introduction of medical abortion using mifepristone and misoprostol in 2001, midwives played a central, double role as advocates and providers.^12^ Medical abortion is now used in 80% of abortion procedures performed in the public sector in Tunisia^13^ and its availability has not caused an increase in the total number of abortions performed each year (Fig. 1).

The country’s new constitution—adopted after the Arab Spring in 2014—recognizes the principles of human rights, including the right to health, reproductive rights, freedom of conscience and religion, respect for privacy, physical and moral integrity, dignity, education, and access to information. However, laws, policies, and practices do not always reflect the new constitution. Furthermore, after the Arab Spring, with growing conservatism and Islamist influence on society in general and on service providers, particularly midwives, we are witnessing the increased stigmatization of abortion, creating barriers that drastically reduce access to legal abortion.

The present study was undertaken as part of a multicountry case study, in collaboration with the WHO, on the inclusion of broader groups of healthcare workers in the delivery of safe abortion care. The aim of the present study was to review the relevant literature on abortion and summarize interviews with key stakeholders to assess the role of midwives in the evolution of abortion‐related care in Tunisia.

## 2 Materials and Methods

A discussion guide for stakeholders was developed for the purposes of the multicountry study and then adapted to each individual country’s local context. For Tunisia, it was translated into French and used to conduct face‐to‐face interviews with eight people selected according to established criteria and representing their organizations.

Interviewees were selected from different organizations and came from different backgrounds: administrators; health professionals, including midwives and doctors involved in different stages of the Tunisian Family Planning Program; and university hospital teachers and midwife trainers. Interviewees represented various profiles and were key members of one of the following institutions: ONFP; a large maternity ward at the La Rabta hospital where several research studies have been conducted; the National Midwives Association; the midwives’ school at the High School for Health Science and Techniques of Tunis, University of Tunis El Manar; Groupe Tawhida Ben Cheikh (GTBC), a local nongovernmental organization (NGO) for women’s health; and Association Tunisienne des Femmes Démocrates (ATFD), a feminist NGO in Tunisia.

The eight interviews were conducted by the same investigator (SH) between August 16 and October 6, 2018. Verbal informed consent was obtained from the participants prior to interview; we specified that they would remain confidential and that interviewees were free to answer the questions that they considered most relevant. The duration of the interviews was between 40 and 90 minutes. The central questions focused around six sections: (1) background and description of the policy in force in Tunisia; (2) strategies, coalitions, collaborations used for implementation; (3) capacity, infrastructure, and resources used for the expansion; (4) personal opinion and user experience; (5) obstacles and facilitators; and (6) control and evaluation.

Interviewees’ answers were transcribed, and the written reports were compiled and grouped by themes according to the six specific sections. The answers provided by each interviewee were compiled and a global analysis of the responses was conducted.

## 3 Results

Although the historical context of abortion in Tunisia is well known, interesting information and details were highlighted and developed by the testimonies of those interviewed.

### 3.1 Sharing of abortion‐related tasks

Since its creation, the ONFP, more than any other department in the Ministry of Public Health, has encouraged task‐sharing of several tasks—including medical abortion—with nonmedical health workers.

Initially and until the end of the 1970s, the most important tasks in the family planning program were defined by specific protocols. From the beginning of the 1980s, midwives were entrusted with contraceptive counseling (including gynecological counseling and examination) owing to limited numbers of physicians and the critical need for providers with good communication skills, especially among the women’s community. Midwives were also allowed to prescribe oral contraceptives, insert intrauterine devices (IUDs), and were trained to assess gestational age by gynecological examination before abortion. From 1997, midwives were also allowed to prescribe antibiotics for STIs in accordance with established guidelines.^14^

With the introduction of medical abortion in 2001, midwives’ responsibilities were extended to include: clinical gynecological examination; prescription of blood tests and/or ultrasound if necessary; counseling for the administration of mifepristone and misoprostol; telephone follow‐up for adverse effects; a control visit two weeks after medical abortion; and postabortion contraception. However, although they perform all tasks in the process of medical abortion, midwives are not permitted to perform other tasks, such as implant placement, ultrasound scans, or written prescription for medical abortion or intrauterine manual vacuum aspiration (MVA); MVA is rarely performed in Tunisia and few midwifes have been trained in MVA.

### 3.2 Services and policies developed for task‐sharing and delegation

The creation of the ONFP in 1974, followed by its training center, enabled the training of qualified and competent health workers, in all tasks described previously. Task‐sharing began informally for nonphysician health professionals represented exclusively by midwifes in the family planning clinics. After several years, these activities were formally incorporated into family planning norms and guidelines (especially contraception and abortion), with a special emphasis on health workers’ tasks. The guidelines were developed in 2006 and amended in 2013.^14^ As the national organization responsible for such programs, the ONFP had to develop, assess, and undertake implementation plans at both regional and national levels. However, the text of the law has not changed since its last edition in 1973.

Task‐sharing and the provision of long‐standing counselling services have been instrumental in strengthening midwives’ knowledge and skills in oral contraception, injectable contraception, IUD insertion, and emergency contraceptive pills; thus, helping them to better understand contraindications and adverse effects.

In the case of medical abortion, midwives participating in research studies and preliminary trials have demonstrated their competence in undertaking all aspects of medical abortion^15^, ^17^ and have been authorized to participate in medical abortion in family planning clinics. Consequently, in ONFP clinics, the medical abortion prescription is generally made by physicians, but midwives carry out the procedure, including recruiting women according to eligibility criteria, counseling them, administering the drugs, monitoring, collecting data, ensuring follow‐up, and prescribing postabortion contraception.

MVA is rarely used in Tunisia, but it has been introduced in the main gynecology service in Tunis and delegated to midwives for two indications: incomplete abortion after medical abortion and surgical abortion in early pregnancies that can be performed outside operating theaters when using MVA with local anesthetic.

Misoprostol administration for cervical dilatation after 12 weeks of pregnancy is delegated to midwives in tertiary hospitals where second‐trimester abortions are performed with a combination of mifepristone and misoprostol or misoprostol alone.

Task‐sharing has also helped reduce regional disparities in human resource allocation due to limited numbers of specialists, gynecologists, and obstetricians in central and southern Tunisia. In practice, nurses and midwives manage first‐trimester medical abortions in these areas, providing administration of misoprostol and follow‐up.

### 3.3 Self‐administered medical abortion

Women’s ability to manage and successfully control their own abortion process has been well demonstrated by research and field practice in Tunisia.^15^, ^16^, ^17^ According to these studies, use of misoprostol at home after mifepristone administration at the clinic was accepted and well managed by women; 80% chose this procedure in the sites where it was proposed.^16^ Furthermore, 40% of the women who benefitted from medical abortion considered that it was necessary to return for the planned follow‐up visit, and the medical records did not indicate any complications.^16^ Through on‐site counseling, women were well informed about the medical abortion process and its potential complications. Additional studies developed in Tunisia showed that women were able to use the semiquantitative urine test to confirm that medical abortion had been successful.^17^

### 3.4 Current obstacles to accessing abortion

Despite the existence of a well‐established task‐sharing policy for health workers, research has identified several barriers to accessing abortion.^18^, ^19^

In the last decade, with new orientations after the Tunisian revolution and the election of a Conservative government in 2012–2014, healthcare providers (mainly midwives and nurses, and then physicians) have gradually developed a conservative attitude toward abortion and have begun to develop a stigmatizing discourse against women and girls who request an abortion. In many cases, women have been denied access to abortion procedures and/or access has been delayed by unnecessary examinations.^18^, ^19^

Regarding providers’ attitudes, a lack of interest among gynecologists or even opposition from obstetrician‐gynecologists has been reported.^19^ The study also revealed ambivalence, if not opposition, from some ONFP administrative or political staff.^20^ These obstacles have never been solved, although the institution itself is the country’s main agency in charge of the regulation and institutionalization of these policies.

### 3.5 Factors influencing providers negative attitudes

Barriers to the provision of reproductive health services were widely discussed and commented upon in the interviews with providers and decision makers, and several recognized that these barriers existed and were influenced by numerous factors:

- Abandonment or lack of political will among Ministry of Health staff members who have conservative opinions that do not support the ONFP program and oppose access to abortion.
- Lack of governance; for example, Medabon (a combination of misoprostol and mifepristone) is legally registered but not available for bureaucratic reasons. Mifepristone is limited by the Central Pharmacy of Tunisia for use only in public services, although the law allows its use in the private sector also (hospitals and private clinics).
- The reluctance and opposition of midwives and physicians who use conscientious objection without respecting its conditions.
- The total disinterest or embarrassment and reluctance of the media and international agencies to deal with issues perceived as taboos.

We were able to draw several conclusions from the interviews:

- The political will to delegate abortion‐related medical tasks to providers other than physicians is essential.
- Sustainability is essential and must be guaranteed by legal texts and guidelines on standards and procedures, legitimizing/enforcing task delegation to value midwives and other providers and prevent exposing them to the risk of prosecution. Access should also be ensured by permanent availability of the products, quality of the services, and the availability of providers whose negative attitudes can be reversed by training in values clarification.
- Research as an evidence tool, and providers’ assessment must accompany all stages of the process.
- Follow‐up and assessment by researchers and practitioners should be put in place from the start of the process and must be maintained.

## 4 Discussion

Tunisia is one of 56 countries in the world where first‐trimester abortion on request is legal, with free access and free services provided in public institutions dedicated to family planning across the country. However, despite the move toward provision of simple and secure abortion procedures, abortion services remain under the responsibility of physicians in authorized institutions, which limits women’s access to them.

The absence of a clearly established policy based on written legal documents with provisions related to the transfer of tasks and implementation strategies has weakened the institution of established programs for decades and worsened abortion access. In addition, the contribution of midwives and other health workers and their effectiveness in provision of medical abortion and contraception have been underestimated and sometimes even neglected. Finally, while an emphasis on human rights has largely been included in training (after the 1994 ICPD), the language used in midwifery training has not been adapted to an appropriate level of knowledge and to cultural context; it also lacks clear descriptions of the concepts of universal human rights and individual freedoms, as well as essential links to universal values. These shortcomings have affected the ability of midwives to understand the concepts of sexual and reproductive rights and to help women fully exercise their rights, including the right to access a safe abortion.

Although medical abortion consulting and delivery facilitators have been trained and are available, their level of clinical competence is not always sufficient. Furthermore, the protocols developed in the guidelines, standards, and procedures are not integrated satisfactorily into ONFP staff’s routine activities. Systematic integration did not occur despite the commitment and availability of several actors. These include: ONFP decision‐makers; trainers and key staff at the ONFP training center (i.e. the team of experts who designed and contributed to the ONFP strategy and its development); and civil society associations that advocate for sexual and reproductive rights and warn about reduced access to contraception and abortion and the need to share tasks with health workers, especially in remote areas.

The obstacles revealed by the interviewees must be addressed and solutions to facilitate these changes are proposed:

1. Legally involve midwives, who are the main providers of all abortion services, but are not fully or legally authorized to provide them (as it is always the physician who delivers the prescription). Training based on Values Clarification for Attitude Transformation (VCAT), consisting of a value/attitude self‐assessment exercise, would greatly contribute to improving the attitude of health staff members and the quality of services.
2. MVA is rarely used in Tunisia; electric aspiration is centralized in public and private hospitals and performed by specialists (gynecologists‐obstetricians), under general anesthesia. Reintroducing MVA—a simple and affordable method of surgical abortion—and delegating the procedure to midwives, would expand the options for abortion health care, including incomplete abortions.
3. Advocate for the availability of medical abortion therapies in private clinics and pharmacies (after 15 years of excellent medical abortion services in the public sector).
4. Allow women to self‐monitor their medical abortions, and thus have a single visit to the clinic. This would be easily achievable, given that 60% of women did not consider it necessary to return for follow‐up control visits as there were no reported failures or complications.^17^

The present study allowed us to develop a structured reflection based on interviews with personnel who had significant experience and solid knowledge of how sexual and reproductive health services were created in Tunisia. The information allowed us to consider proposals for a future strategy aimed at integrating task‐sharing into abortion care and address the barriers to legal and safe abortion access for all women.

A current obstacle to access is the reluctance of providers (physicians and midwives) to provide abortion care. Their views are sometimes more conservative than the current laws.^18^, ^19^ The task‐sharing debate can help decision‐makers and key stakeholders reflect on what can be developed in the context of providers’ professional activities. It can also help politicians and stakeholders understand how to promote greater sharing of tasks to implement best practices within the existing legal framework. Assessment of behaviors and professional attitudes on a regular basis should be introduced as part of regular assessment of the performance of health professionals in public institutions in charge of managing reproductive health in the country.

In conclusion, the following strategies should be addressed as a priority in Tunisia and could be part of more global recommendations:

1. Advocate with the political authorities, decision‐makers, stakeholders, and associations of health professionals and medical staff for task‐sharing in sexual and reproductive health as an effective, safe, and valuable tool to expand access to contraception and risk‐free abortion. It is imperative to establish strong links between them.
2. Improve, support, multiply, and sustain the training of health workers from a human rights perspective, integrating the values of humanism and compassion, highlighting the negative impact of taboos, using appropriate tools such as values clarification training to consolidate motivation and empowerment of midwives. Promote women's empathy and autonomy in training programs.
3. Develop collaboration with other civil society organizations to encourage the recognition of human rights.
4. Include comprehensive contraceptive and abortion programs for health students (physicians, midwives, nurses), and include sex education in school curricula.

## Author Contributions

SH conducted the interviews and drafted the manuscript. HB participated in analysis of the interviews and manuscript writing.

## Conflicts of Interest

The authors have no conflicts of interest.
